# High prevalence of persistent symptoms and reduced health-related quality of life 6 months after COVID-19

**DOI:** 10.3389/fpubh.2023.1104267

**Published:** 2023-02-02

**Authors:** Irma Ahmad, Alicia Edin, Christoffer Granvik, Lowa Kumm Persson, Staffan Tevell, Emeli Månsson, Anders Magnuson, Ingela Marklund, Ida-Lisa Persson, Anna Kauppi, Clas Ahlm, Mattias N. E. Forsell, Josefin Sundh, Anna Lange, Sara Cajander, Johan Normark

**Affiliations:** ^1^Department of Infectious Diseases, Faculty of Medicine and Health, Örebro University, Örebro, Sweden; ^2^Department of Surgical and Perioperative Sciences, Umeå University, Umeå, Sweden; ^3^Department of Clinical Microbiology, Umeå University, Umeå, Sweden; ^4^Department of Infectious Diseases, Karlstad Hospital, Karlstad, Sweden; ^5^Centre for Clinical Research and Education, Region Värmland, Karlstad, Sweden; ^6^School of Medical Sciences, Faculty of Medicine and Health, Örebro University, Örebro, Sweden; ^7^Centre for Clinical Research, Region Västmanland—Uppsala University, Västmanland Hospital Västerås, Västerås, Sweden; ^8^Center for Clinical Epidemiology and Biostatistics, Faculty of Medicine and Health, School of Medical Sciences, Örebro University, Örebro, Sweden; ^9^Department of Community Medicine and Rehabilitation, Umeå University, Umeå, Sweden; ^10^Department of Respiratory Medicine, Faculty of Medicine and Health, Örebro University, Örebro, Sweden

**Keywords:** COVID-19, PACS, long-COVID, post-acute COVID syndrome (PACS), EQ-5D, SARS-CoV-2, Post COVID-19 condition (PCC)

## Abstract

**Background:**

The long-term sequelae after COVID-19 constitute a challenge to public health and increased knowledge is needed. We investigated the prevalence of self-reported persistent symptoms and reduced health-related quality of life (HRQoL) in relation to functional exercise capacity, 6 months after infection, and explored risk factors for COVID-19 sequalae.

**Methods:**

This was a prospective, multicenter, cohort study including 434 patients. At 6 months, physical exercise capacity was assessed by a 1-minute sit-to-stand test (1MSTST) and persistent symptoms were reported and HRQoL was evaluated through the EuroQol 5-level 5-dimension (EQ-5D-5L) questionnaire. Patients with both persistent symptoms and reduced HRQoL were classified into a new definition of post-acute COVID syndrome, PACS+. Risk factors for developing persistent symptoms, reduced HRQoL and PACS+ were identified by multivariable Poisson regression.

**Results:**

Persistent symptoms were experienced by 79% of hospitalized, and 59% of non-hospitalized patients at 6 months. Hospitalized patients had a higher prevalence of self-assessed reduced overall health (28 vs. 12%) and PACS+ (31 vs. 11%). PACS+ was associated with reduced exercise capacity but not with abnormal pulse/desaturation during 1MSTST. Hospitalization was the most important independent risk factor for developing persistent symptoms, reduced overall health and PACS+.

**Conclusion:**

Persistent symptoms and reduced HRQoL are common among COVID-19 survivors, but abnormal pulse and peripheral saturation during exercise could not distinguish patients with PACS+. Patients with severe infection requiring hospitalization were more likely to develop PACS+, hence these patients should be prioritized for clinical follow-up after COVID-19.

## 1. Introduction

Since the start of the Severe acute respiratory syndrome coronavirus 2 (SARS-CoV-2) pandemic, there has been a growing interest in the long-term health consequences of Coronavirus disease 2019 (COVID-19). Previous studies have shown that 49–68% of hospitalized COVID-19 survivors experience persistent symptoms 6–12 months post infection, with fatigue, dyspnea, muscle weakness, and anxiety/depression as the most commonly reported persistent symptoms (1–3). Studies have also reported reduced physical performance in 22–33% of hospitalized COVID-19 survivors, assessed by the one-minute sit-stand test (1MSTST) and six-minute walk test (1, 2), as well as peripheral oxygen desaturation (3). Few studies of COVID-19 sequalae in non-hospitalized patients have so far been published. In a recent large cohort study, 13% of participants experienced persistent symptoms attributable to the infection (4), while another study reported persistent symptoms in 84% of study participants (5). Varying terminology has been used to describe the persistent symptoms and long-term health consequences after COVID-19, such as long-COVID and post-acute COVID-19 syndrome (PACS). The current definition adopted by WHO suggests that “Post-COVID-19 condition occurs in individuals with a history of probable or confirmed SARS-CoV-2 infection, usually 3 months from the onset, with symptoms that last for at least 2 months” (6).

Health-related quality of life (HRQoL) is a term used to describe to what extent different diseases and their treatments affect the physical, emotional, and social health of an individual (7). The existing studies show that a large proportion of COVID-19 patients experience a reduced HRQoL up to 1 year after infection, due to disabilities affecting everyday life (1, 8).

Little attention has been paid to sequalae in patients with mild disease, and the risk factors for long-term health consequences are still largely unknown. Mild COVID-19 is by far the most common disease manifestation and thus generates the majority of PACS cases. In addition, there is paucity of knowledge regarding the benefit of functional exercise tests during clinical follow-ups. The aim of this study was to investigate the prevalence of self-reported persistent symptoms, abnormal physical performance during exercise, and reduced HRQoL, among hospitalized and non-hospitalized COVID-19 patients 6 months after infection. A secondary aim was to explore risk factors for developing long-term sequelae after COVID-19.

## 2. Materials and methods

### 2.1. Study design and study cohort

Data was retrieved from a prospective multicenter cohort study (CoVUm, clinicaltrials.gov ID: NCT04368013) coordinated by Örebro University hospital and University hospital of Umeå, Sweden. Patients were prospectively enrolled between April 2020 and June 2021 from study sites in Örebro, Umeå, Västerås and Karlstad. Hospitalized (≥18 years of age) and non-hospitalized (≥15 years) patients with a positive PCR test for SARS-CoV-2 were eligible for enrolment. Patients hospitalized due to acute COVID-19 infection were enrolled at the Departments of Infectious Diseases and the intensive care units (ICU) at Örebro University Hospital, University Hospital of Umeå, Västerås Central Hospital and Karlstad Central Hospital. Non-hospitalized patients fulfilling the inclusion criteria, were prospectively enrolled using convenience sampling at the infectious diseases' outpatient clinic at University Hospital of Umeå. Exclusion criteria were inability to provide informed consent and inability to read and communicate in Swedish. At Västerås and Karlstad sites, patients hospitalized due to COVID-19 were enrolled at a follow-up visit within 6 months from discharge.

### 2.2. Data collection

Data on disease severity, level of care, clinical and laboratory parameters, and baseline characteristics including comorbidities and medication was collected for each patient. Mortality risk and comorbidity-based disease burden was calculated using the Charlson Comorbidity Index (CCI) (1). Study data was collected and managed using REDCap electronic data capture tools hosted at Umeå University (2).

Follow-up visits were conducted at 2 weeks, 4 weeks, 2 months, 3 months, and 6 months after discharge from hospital or enrolment for hospitalized/non-hospitalized patients, respectively. Patients enrolled at Karlstad and Västerås attended the follow-up protocol from 6 months and onwards. Data was exported from the database on February 20th, 2022.

### 2.3. Outcome measures

Persistent symptoms were assessed with a custom questionnaire containing 15 different symptoms: Cough, dizziness, headache, hyposmia/dysgeusia, experienced impaired memory function, difficulties finding words, mental fatigue, panic attacks, concentration difficulties, sleeping difficulties, nightmares, myalgia, physical fatigue, restless legs and upset stomach, at follow-up visits from 4 weeks until 6 months. In addition, experienced dyspnea was assessed at all follow-up visits with the modified Medical Research Council (mMRC). Dyspnea Scale, ranging from 0 to 4, were 0 corresponds to “dyspnea only during strenuous exercise”, and 4 to “too dyspneic to leave the house or breathless when getting dressed” (3).

Health-related quality of life was measured with the generic health status instrument EuroQol 5-dimension 5-level questionnaire (EQ-5D-5L), covering five dimensions; mobility, usual activities, self-care, pain/discomfort, and anxiety/depression. For each dimension, five levels are presented, ranging from “no problems” to “extreme problems”. EQ-5D-5L also includes the EuroQol Visual Analog Scale (EQ-VAS), which is a visual analog scale for the patients self-assessed overall health, ranging from 0 to 100. The endpoints on the scale are marked as “the worst health you can imagine” and “the best health you can imagine” (4). EQ-5D-5L assessment was added to the follow-up protocol in December 2020, resulting in missing data for the patients enrolled before July 2020.

Due to lack of data on patients' premorbid HRQoL and dyspnea, the following questions were also posed to the study participants: (1) How has your view of the future changed since before your illness? (2) How physically active have you been the past week, compared to before your illness? (3) How has your breathing been the past week, compared to before your illness (Data Sheet 1)?

### 2.4. Functional test of exercise capacity

A 1-minute sit-to-stand test (1MSTST) (5) was performed at each follow-up visit from 4 weeks after enrolment or discharge from hospital. A pulse oximeter was used to record oxygen saturation and heart rate, before and after the test. A decrease in saturation with more than four percent units, and a post-test change of the heart rate with ≤ 0, or more than two standard deviations (SD) from the mean, were considered pathological (6).

### 2.5. Definitions

Disease severity was defined as: Mild (non-hospitalized patients, corresponding to WHO clinical progression scale 1–3 B) and (Severe: hospitalized patients, corresponding to WHO clinical progression scale 4–9) (7).

Impairment in the EQ-5D-5L dimensions was defined as at least moderate difficulties (score ≥ 3) in each dimension. Histogram analysis of the distribution of EQ-VAS-scores was performed, revealing a bimodal distribution with one peak below the score value of 60. We therefore defined a reduced overall health as an EQ-VAS-score ≤ 60. The cut-off for breathlessness was set at mMRC ≥ 1.

We incorporated persistent symptoms and reduced HRQoL into a new definition: PACS+, to enable analysis of the group of patients who, in addition to persistent symptoms, also experienced significant negative consequences in their daily life and/or in their overall health. PACS+ was defined as the prevalence of ≥1 symptom at the 6-month follow-up, together with either moderate (score ≥ 3) difficulties in ≥2 dimensions of EQ-5D-5L and/or self-assessed overall health ≤ 60 in EQ-VAS.

### 2.6. Statistical analysis

Statistical analyses were performed using IBM SPSS statistics (Version 25, IBM Corp., NY, USA), Jamovi (version 2.2.5), GraphPad Prism (version 9.3.1, GraphPad Software, San Diego, CA, USA) and STATA release 17. Groups were compared with *X*^2^-test or Fisher's exact test for categorical variables, and unpaired *T*-test or Mann-Whitney *U*-test for continuous variables, depending on the normality. The normality of continuous variables was tested using the Shapiro Wilks test.

Multivariable Poisson regression with robust standard errors was performed to explore risk factors for developing persistent symptoms, reduced HRQoL and PACS+ and to compare the hospitalized vs. non-hospitalized groups. All analysis comparing the groups were adjusted for age, sex, WHO classified body mass index (BMI), CCI (0 none, 1–2 mild, ≥3 moderate/severe) and smoking status. Effect sizes are presented as relative risk ratios (RR) with 95% confidence intervals (CI). Significance level was set at the 5% level (*p*-value < 0.05). Non-response analyses were performed for the participants who did not complete the EQ-5D-5L questionnaire.

### 2.7. Ethical considerations

The study was conducted in accordance with the Declaration of Helsinki. Written informed consent was obtained from all patients. Ethical approval for the study was granted from Swedish Ethical Review Authority, Uppsala (approval number: 2020-01557).

## 3. Results

### 3.1. Study cohort

**Figure 1:**
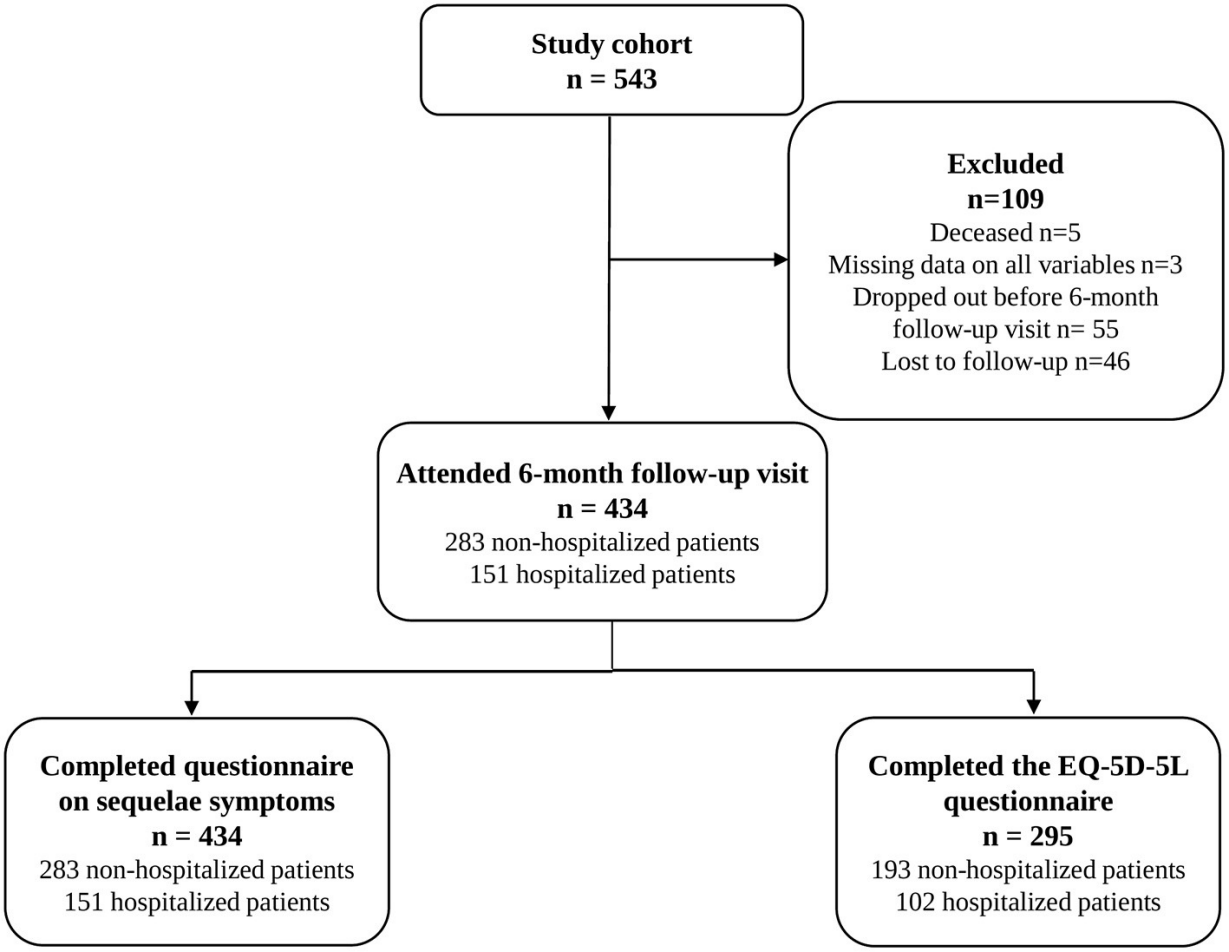
Flowchart of included patients.

At data export, on February 20, 2022, the study cohort comprised 543 patients. Before the 6-month follow up, 55 patients had dropped out of the study and five patients had died. Forty-six patients were lost to follow-up. In total, 434 patients with COVID-19, of which 151 had been hospitalized, attended the 6-month follow-up visit. Data on EQ-5D-5L was available for 295 patients (Figure 1).

### 3.2. Baseline characteristics of the study cohort

Baseline characteristics of the study cohort are presented in Table 1. The median number of days from disease onset to the 6-month follow-up visit for all patients was 192 days. The median age was lower in the non-hospitalized group compared to hospitalized patients (45 vs. 58 years), and a larger proportion of non-hospitalized patients were women (56 vs. 35%). The median BMI of non-hospitalized patients was lower than that for hospitalized patients (25 vs. 30). None of the patients had received any SARS-CoV2 vaccine dose > 14 days before enrolment. All participants were thus considered unvaccinated. Diabetes, hypertension, and cardiovascular disease were significantly more common among hospitalized patients, and a larger proportion were former smokers.

**Table 1:** Demographic and baseline characteristics of all hospitalized and non-hospitalized patients that attended the 6-month follow-up visit.

	**Total (*n* = 434)**	**Non-hospitalized patients (*n* = 283)**	**Hospitalized patients (*n* = 151)**	***p*-value**
Age in years—median (IQR)	48 (36–60)	45 (29–55)	58 (47–65)	< 0.001*
**Sex—n (%)**				
Women	212 (48.8)	159 (56.2)	53 (35.1)	**< 0.001** ^**•**^
BMI—*n*	420	275	145	
BMI—median (IQR)	26.3 (23.4–30.1)	24.8 (22.6–27.5)	30.0 (26.9–33.2)	< 0.001*
< 25 underweight/normal	160 (38.1)	147 (53.5)	13 (9.0)	
25–29 overweight	151 (40.0)	92 (33.4)	59 (40.7)	
≥30 obese	109 (35.9)	36 (13.1)	73 (50.3)	
**Comorbidities—*****n*** **(%)**				
Diabetes	25 (5.8)	7 (2.5)	18 (11.9)	**< 0.001** ^**•**^
Hypertension	86 (19.8)	35 (12.4)	51 (33.8)	**< 0.001** ^**•**^
Cardiovascular disease^a^	31 (7.1)	13 (4.6)	18 (11.9)	**0.005** ^**•**^
Chronic lung disease^b^	74 (17.1)	41 (14.5)	33 (21.8)	0.052^•^
Asthma	67 (15.4)	38 (13.4)	29 (19.2)	0.113^**•**^
Autoimmune disease^c^	24 (5.5)	13 (4.6)	11 (7.3)	0.243^**•**^
Immunocompromised^d^	10 (2.3)	5 (1.8)	5 (3.3)	0.327°
Malignancy^e^	9 (2.1)	3 (1.1)	6 (4.0)	0.071°
CCI—median (IQR)				
0 (0–0)	0 (0–0)	0 (0–1)	< 0.001*
0	328 (75.6)	233 (82.3)	95 (62.9)	
1–2 mild	100 (23.0)	49 (17.3)	51 (33.8)	
≥3 moderate/severe	6 (1.4)	1 (0.4)	5 (3.3)	
Smoking status—*n* (%)				
			**0.001** ^**•**^
Non-smoker	309 (71.2)	218 (77.0)	91 (60.3)	
Current smoker	9 (2.1)	6 (2.1)	3 (2.0)	
Former smoker	116 (26.7)	59 (20.8)	57 (37.7)	
Snuff—*n* (%)	70 (16.1)	48 (17.0)	22 (14.6)	0.519^**•**^
Level of education^f^–*n* (%)				
			0.186^**•**^
Lower	33 (7.9)	18 (6.4)	15 (10.9)	
Medium	191 (45.5)	127 (44.9)	64 (46.7)	
Higher	196 (46.6)	138 (48.8)	58 (42.3)	
Symptoms at onset				
			0.436°
≥1 symptom	418 (96.3)	274 (96.8)	144 (95.4)	
Asymptomatic	16 (3.7)	9 (3.2)	7 (4.6)	
Respiratory support				
			**< 0.001** ^**•**^
No respiratory support	318 (73)	283 (100)	35 (23.2)	
Conventional oxygen therapy only	13 (3)	0 (0)	13 (8.6)	
NIV, HFNC or IMV	103 (23.7)	0 (0)	103 (68.2)	

^a^Ischemic heart disease, congestive heart failure, arrythmias, aortic disease, valvular heart disease, or peripheral arterial insufficiency.

^b^Chronic obstructive pulmonary disease and asthma.

^c^Including rheumatic diseases.

^d^Immune deficiency diseases or immunosuppressive/immunomodulatory medication.

^e^Solid localized tumor, lymphoma, or leukemia.

^f^Level of education missing in 14 patients. The analysis is based on 420 patients. Lower: < 3 years beyond Swedish compulsory school. Medium: 3 years beyond Swedish compulsory school, but no college or university degree. Higher: University or college degree.

^*^Mann-Whitney *U*-test.

^**•**^ *X*^2^-test.

°Fischer's exact test.

Significant *p*-values are written in bold.

*n*, number of patients; BMI, body mass index; CCI, Charlson Comorbidities Index; HFNC, high-flow nasal cannula; IMV, invasive mechanical ventilation; IQR, interquartile range; NIV, non-invasive ventilation.

Twenty-seven patients (27/151, 18% of hospitalized patients) were admitted to the intensive care unit (ICU) during hospitalization. Of all hospitalized patients, 68% received respiratory support with either non-invasive ventilation, high-flow nasal oxygen or invasive mechanical ventilation, and 9% received conventional oxygen therapy only.

The non-response analysis showed that among those who did not complete the EQ-5D-5L questionnaire (*N* = 139), there was a larger proportion of former smokers (Supplementary Table 1). No other significant differences were found in the baseline data.

### 3.3. A majority of both hospitalized and non-hospitalized patients experienced persistent symptoms at 6 months

At 6-month follow-up, persistent symptoms (any symptom) were reported by a majority of both non-hospitalized and hospitalized patients, however it was significantly more common in hospitalized patients (59 of vs. 79%). The most common symptoms overall were physical fatigue, mental fatigue, hyposmia/dysgeusia, and concentration difficulties (Table 2). The most common symptom among non-hospitalized patients was hyposmia/dysgeusia (29%), which was equally common among hospitalized patients (23%).

**Table 2:** Number of hospitalized and non-hospitalized patients (%) with different persistent symptoms.

	**Non-hospitalized patients (*n* = 283)**	**Hospitalized patients (*n* = 151)**	**Adj. RR (*n* = 420)**	***p*-value**
At least one symptom—*n* (%)	168 (59.4)	119 (78.8)	1.23 (1.05–1.43)	**0.011**
**Respiratory symptoms—*****n*** **(%)**				
Cough	19 (6.7)	25 (16.6)	1.64 (0.83–3.23)	0.152
Dyspnea (mMRC ≥ 1)—***n (%)***	• *n* = 236 • 32 (13.6)	• *n* = 130 • 75 (57.7)	3.83 (2.50–5.86)	**< 0.001**
**Neurological symptoms—*****n*** **(%)**				
Dizziness	31 (11.0)	24 (15.9)	1.48 (0.75–2.91)	0.255
Headache	37 (13.1)	37 (24.5)	1.85 (1.06–3.23)	**0.031**
Hyposmia/dysgeusia	81 (28.6)	34 (22.5)	0.71 (0.45–1.12)	0.144
Impaired memory function	50 (17.7)	58 (38.4)	1.92 (1.29–2.85)	**0.001**
Difficulties finding words	57 (20.1)	53 (35.1)	1.50 (1.01–2.25)	**0.045**
Mental fatigue	62 (21.9)	62 (41.1)	1.74 (1.22–2.48)	**0.002**
**Psychiatric symptoms—*****n*** **(%)**				
Panic attacks	31 (11.0)	35 (23.2)	2.54 (1.39–4.65)	**0.002**
Concentration difficulties	54 (19.1)	58 (38.4)	1.95 (1.32–2.90)	**< 0.001**
Sleeping difficulties	54 (19.1)	42 (27.8)	1.27 (0.82–1.97)	0.284
Nightmares	24 (8.5)	24 (15.9)	1.60 (0.77–3.32)	0.209
**Other—*****n*** **(%)**				
Myalgia	23 (8.1)	31 (20.5)	1.54 (0.81–2.94)	0.186
Physical fatigue	55 (19.4)	71 (47.0)	1.98 (1.38–2.84)	**< 0.001**
Restless legs	17 (6.0)	31 (20.5)	3.00 (1.47–6.11)	**0.002**
Upset stomach	33 (11.7)	24 (15.9)	0.99 (0.54–1.83)	**0**.984

Relative risk ratios and *p*-values have been adjusted for age, sex, BMI, CCI, and smoking status. Significant *p*-values are written in bold.

Adj., adjusted; RR, relative risk ratio; EQ-5D-5L, EuroQol 5-level 5-dimension questionnaire; EQ-VAS, EuroQol Visual Analog Scale.

Hospitalization was independently associated with neuropsychiatric symptoms, i.e., the experience of impaired memory function, difficulties finding words, mental fatigue, concentration difficulties, panic attacks, and headache. Hospitalization was also associated with an increased risk of dyspnea (mMRC ≥ 1), restless legs, and physical fatigue (Table 2).

### 3.4. Problems with daily usual activities, physical activity, and pain/discomfort were more common in hospitalized patients

Problems with usual activities, mobility, and pain/discomfort, measured by the EQ-5D-5L questionnaire, were more common in the hospitalized group. However, pain/discomfort and usual activities were the only two EQ-5D-5L dimensions that were independently associated with hospitalization (Table 3). A large proportion of patients reported that they were able to perform less physical activity than prior COVID-19; 30% of non-hospitalized and 55% of hospitalized patients (*p* = 0.005). Of the non-hospitalized patients, 4% experienced moderate to severe breathing impairment compared to before their illness, vs. 23% in the hospitalized group (*p* < 0.001). A large proportion of patients experienced a more negative view of the future compared to before illness (14 vs. 21% of non-hospitalized/hospitalized).

**Table 3:** Results from the multivariable regression analysis on the variables from the EQ-5D-5L questionnaire and the three additional questions added by the research group.

	**Non-hospitalized patients (*n* = 193)**	**Hospitalized patients (*n* = 102)**	**Adj. RR**	***p*-value**
**EQ-5D-5L dimensions—*****n*** **(%)**				
Mobility: problems with walking around	5 (2.6)	15 (14.7)	3.26 (0.92–11.52)	0.067
Personal care: problems with washing or dishing	2 (1.0)	4 (3.9)	1.59 (0.10–24.48)	0.738
Usual activities: problems with usual activity	14 (4.7)	21 (20.6)	**2.31 (1.04–5.17)**	**0.041**
Pain or discomfort	20 (10.4)	26 (25.5)	**2.23 (1.07–4.67)**	**0.033**
Anxiety or depression	16 (8.3)	14 (13.7)	2.10 (0.58–7.59)	0.256
	*n* = 191	*n* = 101		
EQ-VAS ≤ 60—*n* (%)	22 (11.5)	28 (27.7)	**2.11 (1.04–4.29)**	**0.038**
EQ-VAS—mean (±SD)	78 (±15)	72 (±19)	−5.1 (−10.2 to 0.0)^a^	0.052
**Additional questions—*****n*** **(%)**				
Future outlook: changed negatively compared to before illness	27 (14.1)	21 (20.8)	1.43 (0.67–3.02)	0.355
Physical activity: can do less than before illness	57 (29.8)	56 (54.9)	**1.71 (1.18–2.49)**	**0.005**
Breathing: moderately to severely worsened compared to before illness	7 (3.7)	• *n* = 100 • 23 (23.0)	**7.78 (2.94–20.64)**	**< 0.001**
Capacity to work: reduced compared to before illness	• *n* = 176 • 19 (10.8)	• *n* = 40 • 20 (20.0)	0.94 (0.49–1.78)	0.840
PACS+–*n* (%)	22 (11.4)	32 (31.4)	**2.59 (1.30–5.17)**	**0.007**

Relative risk ratios and *p*-value have been adjusted for age, sex, BMI, CCI, and smoking status. Significant *p*-values are written in bold.

^a^Analyzed with linear regression which gives mean difference with 95% CI as association measure.

Adj., adjusted; RR, relative risk ratio; mMRC, modified Medical Research Council.

### 3.5. Hospitalization was the main risk factor for persistent symptoms, reduced overall health, and PACS+

Hospitalization was the main risk factor for experiencing several symptoms and a reduced HRQoL 6 months after infection. Female sex increased the risk of experiencing at least one symptom, especially dyspnea and neuropsychiatric symptoms, such as mental fatigue, concentration difficulties, and experience of impaired memory function (Table 4). Female patients were also at greater risk of experiencing long-term hyposmia/dysgeusia, dyspnea and physical fatigue. Among specific comorbidities, cardiovascular disease was associated with mental fatigue and malignancy with at least one persisting symptom and dyspnea (Table 4).

**Table 4:** Risk factors for developing the most commonly occurring persistent symptoms, reduced overall health ( ≤ 60 in EQ-VAS), and PACS.

	**Outcomes**								

	**At least one symptom (*****n*** = **420)**	**Dyspnea (mMRC** ≥**1) (*****n*** = **357)**	**Physical fatigue (*****n*** = **420)**	**Hyposmia/ dysgeusia (*****n*** = **420)**	**Mental fatigue (*****n*** = **420)**	**Concentration difficulties (*****n*** = **420)**	**Impaired memory function (*****n*** = **420)**	**Reduced overall health**^b^ **(*****n*** = **278)**	**PACS**+ **(*****n*** = **281)**
	**Adj. RR (95% CI)**	**Adj. RR (95% CI)**	**Adj. RR (95% CI)**	**Adj. RR (95% CI)**	**Adj. RR (95% CI)**	**Adj. RR (95% CI)**	**Adj. RR (95% CI)**	**Adj. RR (95% CI)**	**Adj. RR (95% CI)**
Hospitalization	**1.23 (1.05–1.44)**	**3.82 (2.49–5.86)**	**1.98 (1.37–2.86)**	0.72 (0.46–1.12)	**1.72 (1.21–2.45)**	**1.94 (1.32–2.86)**	**1.93 (1.30–2.86)**	**2.38 (1.16–4.90)**	**2.77 (1.36–5.65)**
Age	1.00 (0.99–1.01)	1.01 (0.99–1.02)	1.01 (0.99–1.02)	1.00 (0.99–1.01)	1.01 (0.99–1.02)	1.01 (0.98–1.02)	1.01 (0.99–1.02)	1.00 (0.98–1.02)	0.99 (0.97–1.01)
Female sex	**1**.**21 (1**.**05–1**.**39)**	**1.46 (1.06–1.99)**	**1.36 (1.01–1.83)**	**1.43 (1.01–2.04)**	**1.72 (1.27–2.32)**	**1.47 (1.05–2.05)**	**1.60 (1.15–2.22)**	1.43 (0.84–2.44)	1.37 (0.82–2.28)
**Comorbidities**									
Diabetes	1.12 (0.90–1.39)	0.94 (0.54–1.64)	1.12 (0.70–1.78)	1.03 (0.50–2.08)	1.49 (0.92–2.42)	1.33 (0.79–2.26)	1.29 (0.76–2.20)	1.79 (0.89–3.60)	1.38 (0.68–2.79)
Hypertension	0.99 (0.83–1.18)	1.01 (0.69–1.49)	1.21 (0.84–1.74)	0.92 (0.57–1.48)	0.85 (0.57–1.27)	0.79 (0.49–1.27)	0.91 (0.57–1.43)	1.36 (0.68–2.73)	1.49 (0.76–2.94)
Cardiovascular disease	1.17 (0.98–1.39)	1.05 (0.68–1.63)	1.38 (0.91–2.08)	1.41 (0.83–2.39)	**1.75 (1.13–2.71)**	1.52 (0.92–2.50)	1.30 (0.77–2.21)	0.87 (0.28–2.69)	1.05 (0.39–2.79)
Chronic lung disease	1.00 (0.65–1.53)	1.40 (0.56–3.52)	0.96 (0.28–3.22)	1.52 (0.65–3.56)	1.75 (0.79–3.90)	1.20 (0.41–3.50)	1.58 (0.71–3.50)	2.29 (0.32–16.4)	1.66 (0.18–14.8)
Asthma	1.24 (0.81–1.90)	1.06 (0.40–2.82)	1.56 (0.46–5.29)	0.64 (0.26–1.59)	0.97 (0.44–2.16)	1.33 (0.44–3.97)	1.21 (0.53–2.77)	0.61 (0.08–4.57)	0.88 (0.09–8.12)
Autoimmune disease	1.02 (0.80–1.28)	1.48 (0.97–2.26)	0.75 (0.40–1.38)	1.36 (0.80–2.30)	1.19 (0.73–1.94)	1.10 (0.57–2.11)	1.04 (0.55–1.97)	2.13 (0.98–4.63)	2.14 (0.92–5.00)
Malignancy	**1**.**31 (1**.**14–1**.**50)**	**2.24 (1.27–3.98)**	1.30 (0.58–2.90)	1.12 (0.36–3.48)	0.94 (0.43–2.08)	1.37 (0.70–2.65)	1.00 (0.42–2.37)	1.36 (0.28–6.55)	1.33 (0.36–4.90)
Smoking^a^	**1**.**18 (1**.**03–1**.**36)**	1.18 (0.86–1.62)	0.86 (0.62–1.20)	1.07 (0.74–1.55)	0.93 (0.66–1.32)	1.04 (0.72–1.48)	1.17 (0.80–1.71)	1.05 (0.59–1.88)	0.98 (0.57–1.70)
**BMI**									
< 25 underw/normal	Reference	Reference	Reference	Reference	Reference	Reference	Reference	Reference	Reference
25–29 overweight	0.96 (0.80–1.15)	0.79 (0.49–1.26)	1.07 (0.71–1.60)	1.19 (0.81–1.73)	1.09 (0.75–1.57)	1.24 (0.81–1.88)	1.39 (0.90–2.15)	0.81 (0.37–1.79)	1.24 (0.57–2.69)
≥30 obese	1.02 (0.83–1.25)	1.04 (0.65–1.67)	1.20 (0.79–1.82)	1.12 (0.69–1.81)	0.90 (0.60–1.35)	0.91 (0.57–1.46)	1.00 (0.61–1.63)	0.94 (0.42–2.08)	1.06 (0.46–2.44)

Significant relative risk ratios with a *p*-value < 0.05 are written in bold.

^a^Current or former smoker.

^b^ ≤ 60 in the EQ-VAS.

Adj., adjusted; RR, relative risk ratio; mMRC, modified Medical Research Council; PACS, post-acute COVID-19 syndrome.

Out of the 295 patients that completed the EQ-5D-5L questionnaire, 54 (18%) met the criteria for PACS+ including at least one persisting symptom and reduced HRQoL. Hospitalization was the only variable associated with an increased risk of developing PACS+ with a relative risk ratio of 2.77 (95% CI 1.36–5.65, Table 4).

### 3.6. PACS+ was associated with a lower exercise performance, but not with desaturation or abnormal pulse reaction

**Figure 2:**
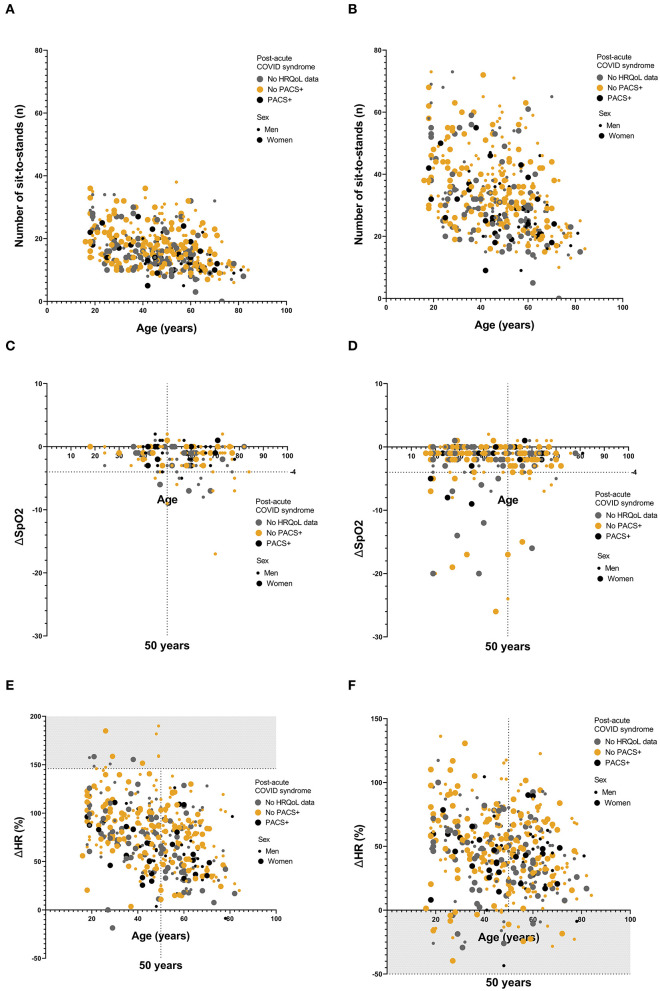
**(A–F)** Results from 1MSTST presented by number of sit-to-stands, oxygen saturation and heart rate. **(A)** Number of sit-to-stands at 30 s for the whole cohort. Age in years presented on the *x*-axis. Numbers presented are the minimum values observed for each study participant between 4 weeks and 6 months. PACS+, black, non-PACS+, yellow and values for patients with no recorded HRQoL data, in gray. Women, large dot; men, small dot. Median values were 14 (range: 5–27) for PACS+, vs. 17 (range: 6–38) for non-PACS+, *p* = 0.021 (Mann-Whitney *U*-test). **(B)** Number of sit-to-stands at 60 s for the whole cohort. Age in years presented on the *x*-axis. Numbers presented are the minimum values observed for each study participant between 4 weeks and 6 months. PACS+, black, non-PACS+, yellow and values for patients with no recorded HRQoL data, in gray. Women, large dot; Men, small dot. Medians: 26 (range: 9–55) for PACS, vs. 33 (range: 10–73) for non-PACS+, *p* = 0.005 (Mann-Whitney *U*-test). **(C, D)** Values of delta-SpO2 after 1MSTST, divided by hospitalized **(C)** and non-hospitalized **(D)**. PACS+, black, non-PACS+, yellow and values for patients with no recorded HRQoL data, in gray. Women, large dot; men, small dot. Age in years presented at *x*-axis, with a dotted line at 50 years. Another dotted line at delta-SpO2 −4, with values on or below this line regarded as pathological. Forty-two participants (15%) had a pathological decrease in oxygen saturation during the test, of which six participants (14%) were also defined as PACS+, (*p* = 0.664) (Fisher's exact test). **(E, F)** Values of delta-Heart rate in percent after 1MSTST, presented as maximum values **(E)** and minimum values **(F)** observed for each study participant between 4 weeks and 6 months. PACS, black, non-PACS+, yellow values for patients with no recorded HRQoL data, in gray. Women, large dot; men, small dot. Age in years presented at x-axis, with a dotted line at 50 years. The Gray dotted areas in the graphs represent pathological values. The limit is set at 0% for minimum values and at +2SD from the mean at maximum values, 146%. Twenty-four participants (8%) had a pathological change in heart rate. Of these, three participants (13%) were also defined as PACS (*p* = 0.779) (Fisher's exact test).

Among the 295 patients that completed EQ-5D-5L questionnaire at 6 months follow up, and thus could be defined as PACS+ (*n* = 54) or non-PACS+ (*n* = 241), 289 (100/289, 35% hospitalized) performed a 1MSTST between 4 weeks and 6 months after study enrolment. Out of these, 12 patients discontinued the test before 60 s, mainly due to pain and or discomfort in lower extremity/extremities. PACS+ patients performed significantly fewer elevations compared to non-PACS+ patients, both at 30 s (14 vs. 17, *p* = 0.021) (Figure 2A), and 60 s (26 vs. 33, *p* = 0.005) (Figure 2B). Forty-two patients (42/289, 15%) had a pathological decrease in oxygen saturation during at least one 1MSTST between 4 weeks and 6 months after COVID-19, but among these only 6 (14%) had PACS+ (Figures 2C, D). Twenty-four patients (24/289, 8%) showed an abnormal change in heart rate during at least one 1MSTST. Out of these, three patients (13%) had PACS+ (Figures 2E, F). There were no significant differences with regards to age, sex, or proportion hospitalized between patients with or without desaturation or pathological heart rate during 1MSTST.

## 4. Discussion

We found that neuropsychiatric symptoms are more common than respiratory symptoms 6 months after COVID-19. Although most patients experienced persistent symptoms, less than one fifth had a significant impact on their HRQoL, with hospitalization being the most important risk factor for long-term sequelae. While numerous studies of symptoms after COVID-19 have been published, most studies either have a small sample size, include only non-hospitalized or hospitalized patients, or lack assessment of HRQoL and/or physical exercise capacity (8). The CoVUm cohort is unique in its design, including both hospitalized patients with severe COVID-19 and a large proportion of non-hospitalized patients with mild disease. Moreover a very low fraction of patients was lost to follow-up, and a detailed follow-up protocol was used, including objective tests of functional exercise capacity. This enabled in-depth analysis of risk factors for persistent symptoms, reduced HRQoL as well as abnormal physical reactions during exercise. Finally, a major strength of the present study is the prospective design which enables a more correct assessment of the long-term effects of this disease in contrast to studies enrolling patients after presentation of suspected PACS-related symptoms.

The high prevalence of neuropsychiatric symptoms in both hospitalized and non-hospitalized patients 6 months after infection was the most striking finding, in particular since the non-hospitalized group was a generally healthy patient cohort upon enrolment. Although previous studies have also shown that a large proportion of COVID-19 patients report long-term problems with memory loss, insomnia, and mental slowness (9, 10), the evidence is conflicting as to whether the symptoms are preexisting, related to the infection, severe disease in general, or to indirect effects of the COVID-19 pandemic (11). Our study cannot establish causality, but the data suggest that neuropsychiatric symptoms occur in all patient groups, even though these potentially disabling symptoms are much more common in hospitalized patients. Recent studies have started to provide possible mechanistic explanations to the impact of COVID-19 on the central nervous system. Douaud et al. revealed important differences in brain structure between COVID-19 patients and matched controls; COVID-19 patients exhibited a reduction in gray matter and global brain size, compared to before illness (12). Rau et al. showed that white matter changes and signs of vasogenic oedema are associated with cognitive impairment during the subacute phase of COVID-19 (13). Recently, white-matter-selective microglial reactivity and increased levels of the proinflammatory chemokine CCL11 leading to impaired hippocampal neurogenesis was suggested as pathophysiological mechanisms behind cognitive impairment (14). Perceived hyposmia/dysgeusia and dizziness were the only neuropsychiatric symptoms that were equally common among non-hospitalized in our study. This finding is consistent with that of previous research, as many studies have reported a high prevalence of experienced hyposmia/dysgeusia (15).

We also show that women had a higher risk for experiencing long-term symptoms in general and, in particular, physical fatigue, mental fatigue, hyposmia/dysgeusia, concentration difficulties, and experience of impaired memory function. These findings are in congruence with previous research both within (16) and outside the COVID-19 research field (17). Multiple explanations, biological and sociocultural, to why women report symptoms at a higher frequency than men, have been proposed. In the case of COVID-19 female sex hormones seem to have an impact on the disease phenotype. Studies investigating this sex-related difference in disease outcome have elucidated that female sex hormones, such as estrogen, may play an important role in protection against severe disease (18). However, in contrast to the protective effect in the acute phase, female sex hormones may partly contribute to the increased risk of persistent symptoms post-infection (19). Further studies on subgroups of patients with different sex hormone levels and outcomes are needed to investigate the biological background to differences in disease phenotype.

Importantly, our study also included assessment of the patients' HRQoL. Since self-reported persisting symptoms are common after COVID-19, 66% in our cohort, the current definition of PACS (at least on persisting symptom) is of limited use to identify patients in need of resource demanding clinical follow-ups and rehabilitation efforts. We therefore added reduced HRQoL to our definition, PACS+, which applied to 18% of the cohort. Hospitalization was the single most important risk factor for PACS+, which indicates that these patients should be prioritized for clinical assessment post-infection. Reduced overall health and problems with pain/discomfort and usual activities were more common among patients with severe disease, which is not uncommon after critical disease regardless of cause (20). In critical care, Thiolliere et al. recently demonstrated that there was no difference in self-reported HRQoL between COVID-19 patients and non-COVID-19 patients 6 months after ICU discharge (11). We used validated instruments, EQ-5D-5L and EQ-VAS, for HRQoL assessment, and our results may be compared to previous Swedish cohort studies. Here, COVID-19 has a negative impact also in the group with mild disease. In a study from 2001 consisting of a cohort with similar age distribution as ours, mean self-rated overall health was 85, compared to 72 and 78 for hospitalized and non-hospitalized patients, respectively, in our study (21). Another more recent Swedish study, where the mean age of the cohort was noticeably higher (64 years), reported a mean EQ-VAS of 76 (22). The impact on HRQoL in mild COVID-19 is supported by recently published data (9).

The inclusion of a validated physical exercise test enabled us to assess abnormal physical reactions to exercise in patients with and without PACS+. A group of patients presented with an abnormal pulse response and/or oxygen desaturation during the 1MSTST. These test results are indicative of autonomic dysfunction, a phenomenon that has previously been described after COVID-19 (23), but to our knowledge not in relation to reduced HRQoL. In the present study, these physical signs were not significantly associated with PACS+, nor with hospitalization or a certain sex. However, autonomic dysfunction has also been associated with objective functional limitations, not with subjective symptoms or limitations (24). We argue that this may be a reason as to why pulse and oxygen saturation reaction, consistent with autonomic dysfunction, were not associated with PACS+ as defined here. A large proportion of patients reported that they had a lower physical performance level after COVID-19 compared to before the illness. This indicates that exercise capacity may be affected, but not perceived as reduced HRQoL by the patient. The underlying pathophysiology and implication of autonomic dysfunction needs to be studied further to distinguish any characterizing factors. We acknowledge that our study has a number of limitations, including the lack of participants' baseline data of HRQoL, symptoms and physical exercise capacity, lack of information on the number of patients that declined participation, and the exclusion of patients with pronounced cognitive dysfunction, and/or inability to read and communicate in Swedish. These patients potentially differ from others in terms of comorbidities and socioeconomic status, which are factors that may affect the long-term health outcomes. In our cohort, 46 patients were lost to follow-up and 139 did not complete the EQ-5D-5L questionnaire, which may result in a minor bias. In addition, our study was not powered to study effects of interventions, for example early antiviral treatment or specific rehabilitation programs, on incidence and severity of long-term health sequalae after COVID-19.

In conclusion, long-term symptoms after COVID-19 were commonly reported in this longitudinal, prospective, multicenter COVID-19 study. We identified that hospitalization due to severe COVID-19 was the single most important independent risk factor for developing clinically relevant long-term health consequences. Less than a third of the hospitalized patients experienced a significantly reduced quality of life. Our aim was to identify patients who experience significant negative consequences in their daily life and need to be prioritized for follow-up. Thus, we suggest an adjustment of the definition of PACS by adding low HRQoL measured by EQ-5D-5L or EQ-VAS.

Our findings, and the suggested PACS+ definition, may hopefully guide optimization of algorithms for clinical long-term follow-up after COVID-19.

## Data availability statement

The datasets presented in this article are not readily available because ethical approval does not support publication of the entire dataset of the study cohort. Requests to access the datasets should be directed to johan.normark@umu.se.

## Ethics statement

All procedures performed in studies involving human participants were in accordance with the ethical standards of the institutional and/or national research committee and with the 1964 Helsinki Declaration and its later amendments or comparable ethical standards. Informed consent was obtained from all individual participants involved in the study.

## Author contributions

AE, CA, JN, SC, AK, and AM: conceptualization and methodology. AK, I-LP, SC, EM, ST, and IM: data collection. JN, SC, and AE: supervision. AE, CA, JN, MF, SC, ST, EM, and IM: resources. AE, IA, LK, CG, SC, and AM: formal analysis. AE, IA, LK, CG, and SC: data curation. IA and SC: writing—original draft. AE, IA, LK, CG, AL, CA, JN, JS, MF, SC, ST, AK, I-LP, EM, IM, and AM: writing—review and editing and visualization. CA, JN, MF, SC, ST, and EM: funding acquisition. AL, JN, SC, ST, and EM: project administration. All authors contributed to the article and approved the submitted version.
