# Supplementary material for: High prevalence of persistent symptoms and reduced health-related quality of life 6 months after COVID-19
Source: Front Public Health. 2023 Feb 2;11:1104267. doi: 10.3389/fpubh.2023.1104267 (PMC9932930; doi:10.3389/fpubh.2023.1104267)
Supplement: Supplementary file 1 [file Table_1.pdf]

frontiers

Supplementary Material

Supplementary Table 1. Demographic and baseline characteristics in patients that completed the EQ-5D-5L questionnaire, compared to patients that did not. Significant p-values are written in bold.

	**Patients that did not complete EQ-5D-5L (n = 139)**	**Patients that did complete EQ-5D-5L (n = 295)**	**p-value**
Age in years – median (IQR)	48 (34-59)	49 (36-61)	0·279*
Sex – n (%)			0·665•
Women	70 (50·4)	142 (48·1)	
BMI – median (IQR)	26·1 (23·0-30·0)	26·4 (23·5-30·1)	0·705*
***Comorbidities* - n (%)**			
Diabetes	4 (2·9)	21 (7·1)	0·077•
Hypertension	29 (20·9)	57 (19·3)	0·707•
Cardiovascular disease[a]	11 (7·9)	20 (6·8)	0·669•
Chronic lung disorder[b]	23 (16·5)	51 (17·3)	0·848•
Asthma	21 (15·1)	46 (15·6)	0·896•
Autoimmune disease[c]	10 (7·2)	14 (4·7)	0·298•
Immunosuppression[d]	4 (2·9)	6 (2·0)	0·733°
Malignancy[e]	1 (0·7)	8 (2·7)	0·283°
CCI– median (IQR)	0 (0-0)	0 (0-1)	0·250*
***Smoking status* - n (%)**			**0·001•**
Non-smoker	112 (80·6)	197 (66·8)	
Smoker	5 (3·6)	4 (1·3)	
Former smoker	22 (15·8)	94 (31·9)	
***Snuff* - n (%)**	26 (18·7)	44 (14·9)	0·317•
***Level of education*[f] - n (%)**			0·607•
Lower	8 (6·0)	25 (8·7)	
Medium	63 (47·0)	128 (44·8)	
Higher	63 (47·0)	133 (46·5)	

[a] Ischaemic heart disease, congestive heart failure, arrythmias, aortic disease, valvular heart disease or peripheral arterial insufficiency.

[b] Chronic obstructive pulmonary disease, asthma and other pulmonary disorders.

[c] Including rheumatic diseases.

[d] Immune deficiency diseases or immunosuppressive/immunomodulatory medication.

[e] Solid localised tumour, lymphoma, or leukaemia

[f] Level of education missing in 14 patients. The analysis is based on 420 patients. Lower: Less than three years beyond Swedish compulsory school. Medium: Three years beyond Swedish compulsory school, but no college or university degree. Higher: University or college degree.

* Mann-Whitney U-test

• X^2-test

° Fischer's exact test

Abbreviations: BMI, Body Mass Index; CCI, Charlson Comorbidities Index; IQR, interquartile range.
