# Supplementary material for: High prevalence of persistent symptoms and reduced health-related quality of life 6 months after COVID-19
Source: Front Public Health. 2023 Feb 2;11:1104267. doi: 10.3389/fpubh.2023.1104267 (PMC9932930; doi:10.3389/fpubh.2023.1104267)
Supplement: Supplementary file 2 [file Data_Sheet_1.pdf]

Data sheet

Table of the additional questions added to the EQ-5D-5L questionnaire comparing the current health status to the pre-COVID-19 infection status.

Question	Answer options			
1. How has your view of the future changed since before illness?	Has not changed at all	Changed in a positive way	Changed in a negative way	
2. How physically active have you been the past week, compared to before your illness?	Same as before	More than before	Less than before	
3. How has your breathing been the past week, compared to before your illness?	Same as before	Slightly worsened	Moderately worsened	Severely worsened
